# The Expression of Netrin-1 in the Thymus and Its Effects on Thymocyte Adhesion and Migration

**DOI:** 10.1155/2013/462152

**Published:** 2013-11-28

**Authors:** Xiao-Kai Guo, Yuan-Feng Liu, Yu Zhou, Xiu-Yuan Sun, Xiao-Ping Qian, Yu Zhang, Jun Zhang

**Affiliations:** Department of Immunology, Key Laboratory of Medical Immunology (Ministry of Health), School of Basic Medical Sciences, Peking University Health Science Center, No. 38 Xueyuan Road, Beijing 100191, China

## Abstract

Netrin-1, a known axon guidance molecule, being a secreted laminin-related molecule, has been suggested to be involved in multiple physiological and pathological conditions, such as organogenesis, angiogenesis, tumorigenesis, and inflammation-mediated tissue injury. However, its function in thymocyte development is still unknown. Here, we demonstrate that Netrin-1 is expressed in mouse thymus tissue and is primarily expressed in thymic stromal cells, and the expression of Netrin-1 in thymocytes can be induced by anti-CD3 antibody or IL-7 treatment. Importantly, Netrin-1 mediates the adhesion of thymocytes, and this effect is comparable to or greater than that of fibronectin. Furthermore, Netrin-1 specifically promotes the chemotaxis of CXCL12. These suggest that Netrin-1 may play an important role in thymocyte development.

## 1. Introduction

The thymus is the site for T-cell development [1]. Bone-marrow-derived progenitors enter the thymus and follow a well-defined differentiation program to complete their maturation [2]. During this process, developmental thymocytes undergo oriented migration throughout the various anatomical niches within the thymic lobules. Eventually, mature T cells export from the thymus to the periphery [1, 3, 4]. Multiple molecules are well documented to play essential roles in guiding the migration of thymocytes, including chemokines, integrins, sphingolipids, cytokines, and hormones [5–9].

The main chemokine expressed in the thymus is CXCL12 (SDF-1*α*), which is secreted by thymic epithelial cells (TEC) located in the subcapsular and medullar regions. CXCL12 preferentially attracts immature CD4^−^CD8^−^ (DN) and CD4^+^CD8^+^ (DP) cells. Accordingly, the corresponding specific receptor CXCR4 is mainly expressed in these stages of thymocyte development. CXCL12/CXCR4-driven signaling is required for proper localization of early progenitors within the cortex. Thymus-specific deletion of CXCR4 *in vivo* led to failed cortical localization of these progenitors, together with arrest of developmental process [10, 11]. In addition, the chemokines CCL25 (TECK) and CCL22 (macrophage-derived chemokine) mediate chemotaxis of immature thymocytes, whereas CCL19 and CCL21 mainly exert chemotactic effects on CD4SP or CD8 SP thymocytes [12, 13].

The extracelluar matrices (ECM), which includes type I, type III, and type IV collagen, galectin, laminin, and fibronectin, are all important mediators for thymocyte migration. These molecules either promote adhesion or deadhesion or chemoattraction of the thymocytes to the microenvironment [14, 15].

Netrin-1, a secreted laminin-related molecule, was originally identified as an important guidance molecule in the nervous system [16]. Varied receptors of Netrin-1 have been reported, such as those deleted in colorectal cancer (DCC) and neogenin, the UNC5 family receptors Unc5a, Unc5b, Unc5c, and Unc5d, A2b, and integrin *α*6*β*4 [17–19]. Netrin-1 exerts either chemoattraction or chemorepulsion effect on its target cells depending on the receptors they have on their cell membrane. For example, neurons with chemoattractive Netrin-1 receptors DCC or neogenin are attracted by a diffusible gradient of Netrin-1 secreted at the midline. However, coexpression of the chemorepulsive Netrin-1 receptor UNC5H2 (Unc5b) with DCC leads to a conversion of attractive force to repulsive force mediated by Netrin-1 [20]. Increasing the amount of evidence has suggested that Netrin-1 and its receptors play important roles outside of the nervous system. They have been reported to be involved in organogenesis, angiogenesis, and tumorigenesis [21–23]. Recently, it has been shown that Netrin-1 is an important negative regulator of inflammation by inhibiting leukocyte migration and attenuating inflammation-mediated tissue injury [24, 25].

Apart from the inhibition of leukocyte migration, the function of Netrin-1 in thymocyte development is not elucidated. In this study, we therefore investigated whether Netrin-1 is expressed in the thymus of mice and its effects on thymocyte behavior. For the first time, we identified that Netrin-1 was expressed in mouse thymus and was primarily expressed in thymic stromal cells, being induced in thymocytes after being stimulated with anti-CD3 or IL-7. We also verified that Netrin-1 could mediate cell adhesion and potentiate the chemotaxis for chemokine CXCL12. These results suggest that Netrin-1 may also be an important mediator, being involved in thymocyte development.

## 2. Materials and Methods

### 2.1. Mice

C57BL/6 mice were bred in the animal breeding facility at Peking University Health Science Center under specific pathogen-free conditions. The experimental procedures on use and care of animals had been approved by the Ethics Committee of Peking University Health Science Center.

### 2.2. Antibodies and Reagents

Anti-CD8 (3.155) and anti-CD4 (GK1.5) were prepared from hybridomas obtained from the American Type Culture Collection. Purified anti-CD3 (145-2c11); fluorochrome-conjugated Abs against CD4 (RM4-5), CD8 (53-6.7), and isotype control Abs were purchased from BD Pharmingen. Recombinant mouse Netrin-1, IL-7 were purchased from R&D Systems (Minneapolis, MN, USA). The CXCR4 chemokine ligand, CXCL12 (SDF-1), and CCR7 chemokine ligand, CCL19 (MIP-3ß), were purchased from Peprotech (London, UK). Function-blocking mAb to *α*6*β*4 (S3-41) was kindly provided by Dr. Vito Quaranta (The Scripps Research Institute).

### 2.3. Isolation of Subsets of DN DP CD4SP CD8SP Thymocytes

Single-cell suspensions of thymocytes were treated with anti-CD8 (3.155) and/or anti-CD4 (GK1.5) mAb and complement (guinea pig sera) to remove relative cells. After two cycles of killing and removal of dead cells by density centrifugation, viable cells were stained with CD4-APC and CD8-APC-Cy7 (53-6.7) and subjected to cell sorting (FACSAria). The purity of individual sorted thymocyte subgroups was 97–99% as analyzed by FACS.

Freshly isolated thymocytes were cultured in the presence of IL-7 (10 ng/mL) for 12 h. Cells were harvested and mRNA extraction was performed to examine Netrin-1 and Unc5b mRNA expression. To induce anti-CD3 engagement, thymocytes were plated in the presence of coated anti-CD3 antibody (5 *μ*g/mL) or uncoated wells during 3–24 h at 37°C. Thymocytes were harvested at different times and used to extract mRNA to detect Netrin-1 and Unc5b mRNA by quantitative real-time PCR.

### 2.4. Isolation of Thymic Stromal Cells

Thymic tissues were first minced to allow release of thymocytes and then were incubated in 15 mL RPMI 1640 on ice for 15 min. The resulting thymic fragments were further separated with a 2 mL borosilicate glass pipette in 10 mL of the fresh RPMI 1640. Medium was changed twice, allowing thymic fragments to settle each time. Thymic fragments were incubated in 5 mL of 0.125% collagenase P (Roche) with 0.1% DNase I (Ambion) in RPMI 1640 at 37° four times for 15 min, each time in the fresh medium supplemented with enzymes with gentle pipetting every 5 min. Cells were collected during enzymatic digestions, washed with RPMI 1640, and used for Percoll isolation. Stromal cells' layers were collected for further studies.

### 2.5. RNA Preparation and Real-Time PCR

RNA was isolated with the TRIzol reagent (Invitrogen) and used as template for cDNA synthesis. Quantitative PCR was performed on an iCycler sequence detection instrument (Bio-Rad) using iQ SYBR Green Supermix (Bio-Rad, Hercules, CA, USA).

The primer sets used were Netrin-1, forward: AAGCCTATCACCCACCGGAAG, reverse: GCGCCACAGGAATCTTGATGC; Unc5a, forward: ATCCCTAACACAGGAATCAGC, reverse: CTAACGATAGGACTCAGCAGG; Unc5b, forward: TGGATCTTTCAGCTCAAGACCCAG, reverse: AAGATGGCCAGCTGGAGCCG; Unc5c, forward: GATGAAACCTCTGGTCTAATTGTG, reverse: CCTTCCGACTCTTCGTAGTG; Unc5d, forward: GTGAACATCTTCGTATCCGT, reverse: TTCTCAATGCCTCTCCTACTC; DCC, forward: CTCTTCACAGGATTGGAGAAAGGC, reverse: GAGGAGGTGTCCAACTCATGATG; neogenin, forward: CGCTACCTTTGAATTAGTTCCT, reverse: GATGATGTAACCTGTAATCTTGCC; DSCAM, forward: CTTTGCGCGTTATGATCCT, reverse: GTGGTGTCGATACTGATG; A2b, forward: ACGTGGCCGTGGGACTC, reverse: GCAGAAGCCCAAGCTGATG; integrin *α*6, forward: GGGACCTTGTACACGGATTGA, reverse: TGGACCTTGGCTCTGAACAGT; integrin *β*4, forward: AGACATGAGGCCCGAGAAACT, reverse: GAATTCTTCCACATTCTCCGTTAAG. The amount of mRNA expression was normalized to the *β*-actin signal amplified in a separate reaction (forward primer: AGAGGGAAATCGTGCGTGAC; reverse: CAATAGTGATGACCTGGCCGT).

### 2.6. Immunofluorescent Staining

Frozen sections (5 *μ*m) of thymus were prepared by cryostatic sectioning of tissues embedded in OCT compound. Sections were fixed with cold acetone, air-dried, washed in PBS, and blocked with 10% normal goat serum in PBS-0.1% BSA. After incubation with optimal dilutions of rabbit anti-Netrin-1 Abs (YT3042; ImmunoWay) and anti-Keratin 8 (Troma-I-c, DSHB, Iowa City, IA, USA) Abs overnight at 4°C, the slides were probed with FITC-conjugated goat anti-rabbit Ig and PE-conjugated goat anti-rat Ig for 60 min at 37°C. Nuclei were stained with Hochest 33258 (Sigma). Analyses were conducted by confocal microscope.

### 2.7. Enzyme-Linked Immunosorbent Assay of Netrin-1

96-well plates (Corning) were coated with rabbit anti-Netrin-1 Abs (100 *μ*L per well; 1 *μ*g/mL in 0.5% BSA in PBS). Each well was blocked with blocking solution containing 5% BSA (Fisher Scientific) in PBS (350 *μ*L per well). Standards made with recombinant Netrin-1 protein or conditioned medium (250 *μ*L) were added to the coated plates, followed by incubation for 16 h at 4°C plus 2 h at 37°C. After three washes with 0.5% BSA in PBS, goat antibody to Netrin-1 (100 *μ*L per well; 1 *μ*g/mL in 0.5% BSA in PBS; R&D Systems) was added to each well, followed by incubation for 30 min at 37°C. After washing, each well was incubated for 30 min at 37°C with horseradish peroxidase-conjugated rabbit antibody to goat (100 *μ*L per well; 1 : 2,000 dilution in blocking solution; Sigma-Aldrich). After extensive washing, plates were incubated for up to 20 min at 22°C with the peroxidase substrate solution o-phenylenediamine dihydrochloride (200 *μ*L per well; SIGMAFAST OPD tablets; Sigma-Aldrich). Reactions were stopped by the addition of 3 M HCl (50 *μ*L per well) and absorbance at 492 nm was measured with a Model 550 microplate reader (Bio-Rad).

### 2.8. Thymocyte Stimulation

Freshly isolated thymocytes were cultured in the presence of IL-7 (10 ng/mL) for 12 h. Cells were harvested and mRNA extraction was performed to examine Netrin-1 and Unc5b mRNA expression. To induce anti-CD3 engagement, thymocytes were plated in the presence of coated anti-CD3 antibody (5 *μ*g/mL) or uncoated wells during 3–24 h at 37°C. Thymocytes were harvested at different times and used to extract total RNA to detect Netrin-1 and Unc5b mRNA by quantitative real-time PCR.

### 2.9. Thymocyte Binding Assays

96-well culture plates (Corning) were coated with various proteins overnight. Thymocytes were added to the protein-coated plates and incubated for 30 min at 37°C in the absence or presence of integrin *α*6*β*4 blocking reagent. Unattached cells were removed by gentle pipetting, and wells of the plates were washed three times with MEM. Attached cells were recovered with phosphate-buffered saline (PBS) containing EDTA, and adhered thymocytes were counted.

### 2.10. Chemotaxis Assay

Thymocyte migratory activity was assessed *ex vivo* in 5 *μ*m pore size Transwell plates (Corning). Membrane inserts were coated with BSA or fibronectin for 1 h at 37°C, followed by 1 h of blocking with 1% BSA. Cell migration was measured in the absence and presence of chemotactic agents, including CXCL12, CCL19, and with these chemotactic agents plus recombinant mouse Netrin-1 (100–500 ng/mL). Thymocytes (2.5 × 10^6^) were plated in the upper chamber in 100 *μ*L of 0.5% BSA/RPMI and 600 *μ*L of 0.5% BSA/RPMI were added to the lower chamber. After 3 h, cells that migrated into lower chambers were removed, counted, and phenotyped for the detection of CD4 and CD8. A quantitation of the chemotaxis was denoted as chemotactic index. It was calculated by dividing the number of migrating cells in the treated groups by the number of migrating cells in the control group.

### 2.11. Flow Cytometric Analysis

Cells were stained with CD4-APC and CD8-APC-Cy7. Appropriate isotype-matched Abs were included for compensation adjustment. Data acquisition and analysis were performed on FACSCalibur using the Cellquest software (BD Bio-Sciences). Dead cells were excluded on the basis of low forward-light scatter (FSC) and propidium staining.

### 2.12. Statistical Analysis

Student's *t*-test for paired data and Kruskal-Wallis test for nonparametric data were performed using GraphPad Prism software (GraphPad Software). Differences were considered significant when *P* values were <0.05.

## 3. Results

### 3.1. Expression of Netrin-1 and Its Receptors in the Thymus

**Figure 1:**
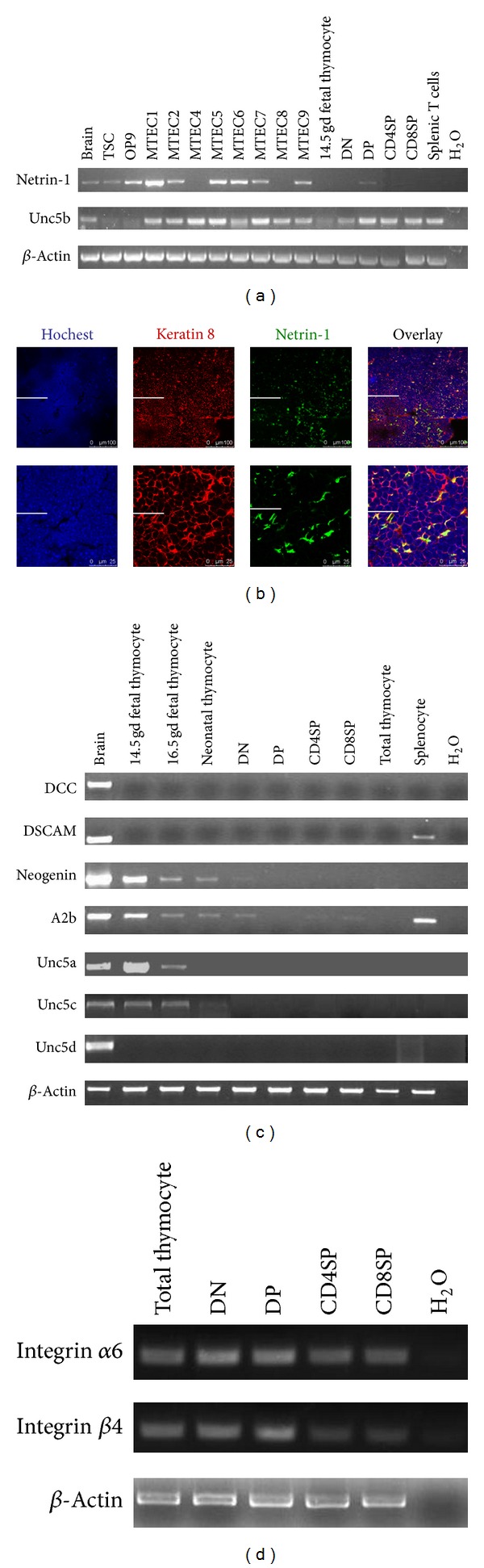
Expression of Netrin-1 and the corresponding receptors in the mouse thymus and thymic cell types. (a) RT-PCR detection of Netrin-1 and Unc5b mRNA expression in the indicated cells. (b) This depicts a section of mouse thymus for the immunofluorescent staining of Keratin 8 (red) and Netrin-1 (green). The microscopic field shows that both molecules are largely colocalized in the thymus. Bars in the above and bottom panels are 100 *μ*m and 25 *μ*m, respectively. ((c) and (d)) RT-PCR detection of other receptors' mRNA expression in the indicated cells. Brain: positive control; TSC: thymic stromal cell; OP9: bone-marrow-derived stromal cell; MTEC: medullary thymic epithelial cell; DN: double negative thymocyte; DP: double positive thymocyte; CD4SP: CD4 single positive thymocyte; CD8SP: CD8 single positive thymocyte; H_2_O: negative control.

In an attempt to identify whether Netrin-1 plays a significant role in thymocyte development, first we investigated whether Netrin-1 or its cognate receptors were expressed in the thymus. By reverse transcription (RT) PCR, it was shown that Netrin-1 was expressed by freshly isolated thymic stromal cells (TSC) as well as multiple thymic epithelial cell lines (Figure 1(a)). Although it was weak, the expression of Netrin-1 in double positive (DP) thymocytes was still detectable (Figure 1(a)). Furthermore, to topologically localize the Netrin-1 expressing cells in the organ, we conducted a double immunofluorescence staining for Netrin-1 in combination with Keratin 8 on cryosections from adult thymus. As shown in Figure 1(b), Netrin-1 showed a broad distribution throughout the thymus, mainly colocalized with Keratin-8-positive cells. However, keratin-8-negative cells were also stained with the anti-Netrin-1 antibody.

We also detected the expression of Netrin-1 receptors. Although DCC, DSCAM, and Unc5d were not detected in the thymus (Figure 1(c)), the expression of neogenin, A2b, Unc5a, and Unc5c was found only in fetal thymocytes or the thymocytes in early developmental stage (Figure 1(c)). Integrin subunit alpha 6 or beta 4 or UNC5 receptor Unc5b represented the main receptors expressed in thymocytes for Netrin-1, which were expressed by four main subsets of thymocytes (Figures 1(a) and 1(d)). Therefore, Netrin-1 or its cognate receptors were expressed in the thymus by thymocytes and/or thymic epithelial cells. These suggest that Netrin-1 may play a role in thymocyte migration and have a potential role in thymocyte development.

### 3.2. Expression of Netrin-1 by Thymocytes Is Inducible

**Figure 2:**
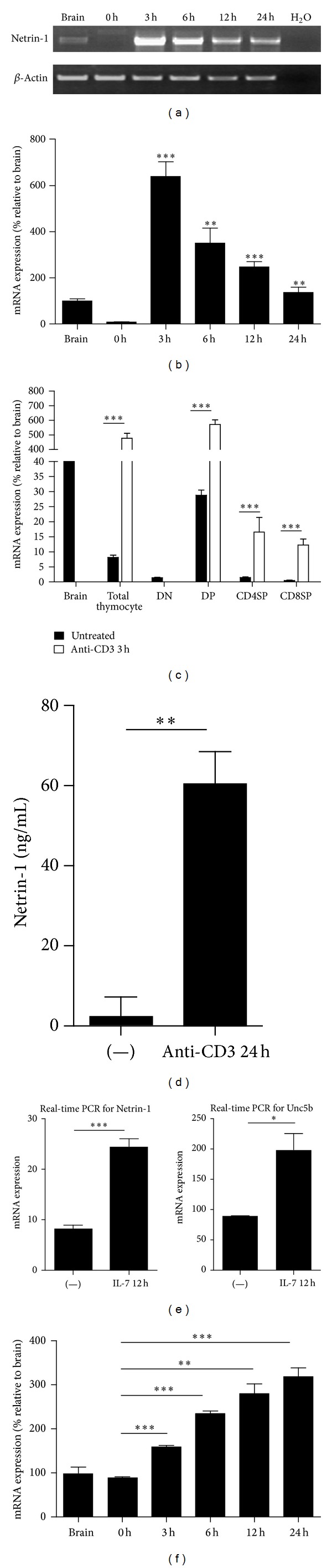
Anti-CD3 and IL-7 stimulation upregulates Netrin-1 and Unc5b expression on thymocytes. ((a) and (b)) RT-PCR showing an increase in Netrin-1 gene expression on thymocytes following anti-CD3 stimulation. Histograms represent the Netrin-1/actin ratio. (c) Real-time PCR showing Netrin-1 expression on thymocyte subsets, DN, DP, CD4SP, and CD8SP before and after anti-CD3 stimulation. (d) Enzyme-linked immunosorbent assay of Netrin-1 in conditioned medium treated with anti-CD3 (5 *μ*g/mL). (e) Real-time PCR showing Netrin-1 and Unc5b expression by IL-7-treated (12 h) or untreated total thymocytes. (f) Real-time PCR showing an increase in Unc5b gene expression on thymocytes following anti-CD3 stimulation. Data shown in each image are representative of three independent experiments. NS: not significant; *P < 0.05; **P < 0.01; ***P < 0.001.

It is known that TCR signaling and IL-7 play crucial role in thymocyte development [26, 27]. As mentioned above, we have shown that Netrin-1 was expressed by fresh thymic stromal cells and multiple stromal cell lines. However, the expression of Netrin-1 in thymocytes was much lower in comparison to stromal cells. Then, we tried to verify if the expression of Netrin-1 in thymocytes could be dynamically regulated. First of all, the thymocytes were stimulated with anti-CD3 antibody. As we predicated, anti-CD3 stimulation could upregulate the expression of Netrin-1 and the expression of Netrin-1 peaked at 3 h after stimulation; then the expression level declined with time (Figure 2(a)). This phenomenon was further confirmed by quantitative real-time PCR (Figure 2(b)). In order to identify whether the upregulation of Netrin-1 in response to anti-CD3 in thymocytes is subset dependent, we obtained DN, DP, CD4SP, and CD8SP thymocytes subsets by cell sorting and these specific thymocytes subsets were stimulated with anti-CD3 antibody for 3 hours. With the exception of DN subset which did not respond to anti-CD3 stimulation, DP, CD4SP, and CD8SP all expressed high levels of Netrin-1 after anti-CD3 stimulation (Figure 2(c)). We also further confirmed the upregulation of Netrin-1 in response to anti-CD3 with ELISA assay. The protein level of Netrin-1 in the supernatants of thymocytes was also upregulated by anti-CD3 stimulation (Figure 2(d)). It is known that IL-7 plays an important role in early T-cell development. Therefore, we investigated the effect of IL-7 on expression of Netrin-1 in thymocytes, and the result demonstrated that Netrin-1 was clearly upregulated by IL-7 (Figure 2(e)). Importantly, Netrin-1 receptor Unc5b was also upregulated by anti-CD3 or IL-7 (Figures 2(e) and 2(f)). These indicated that Netrin-1 or its receptor Unc5b expression was dynamically regulated by thymocyte development signaling, suggesting that Netrin-1 may play a role in thymocyte development.

### 3.3. Netrin-1 Mediates Thymocyte Adhesion

**Figure 3:**
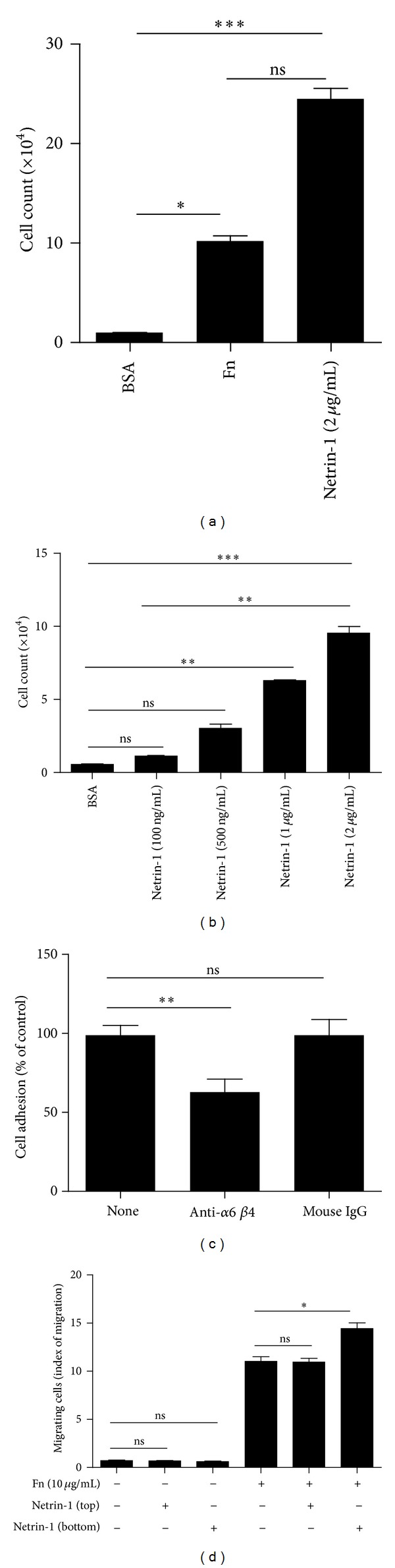
Adhesive effect of Netrin-1 on thymocytes. (a) Quantification of thymocyte adhesion to plate coated with BSA, fibronectin (Fn), or Netrin-1. (b) The inserts of Transwell were coated with different concentrations of Netrin-1, and thymocytes were plated in the upper chamber of Transwell. Histograms represent the number of migrating thymocytes recovered in the lower chamber. (c) Thymocytes were incubated with the function-blocking anti-integrin antibody for 30 min prior to plating on Netrin-1. Cells were allowed to attach for 30 min, then washed, and quantified as described in Materials and Methods. (d) In the presence of Netrin-1 (500 ng/mL), thymocytes were plated in the upper chamber of Transwell under cell migration driven by fibronectin (10 *μ*g/mL). Histograms represent the number of migrating thymocytes in the lower chamber. Data shown in each image are representative of three independent experiments. ns: not significant; *P < 0.05; **P < 0.01; ***P < 0.001.

Further to our identification of the expression of Netrin-1 and its receptors in the thymus, we tried to find the exact functions of Netrin-1 in the thymus. Because we already know that Netrin-1 is a laminin-related molecule, we first tried to address whether Netrin-1 has the same function as other extracellular matrices. Fibronectin is the main extracellular matrix that mediates thymocyte adhesion and chemoactivity. By the cell adhesion assay with Netrin-1 or fibronectin-coated plates, respectively, we found that Netrin-1 mediated thymocyte adhesion which was comparable to or even stronger than what fibronectin did (Figure 3(a)). And this effect was also confirmed by Transwell assay, in which the inserts were coated with Netrin-1. It was dose dependent (Figure 3(b)). Next, we tried to determine the receptors involved in Netrin-1-mediated thymocyte adhesion. As we have mentioned above, integrin subunit alpha 6 or beta 4 or UNC5 receptor Unc5b represented the main receptors expressed in thymocytes for Netrin-1. Unc5b receptor was reported to be important for Netrin-1-induced chemorepulsive effects [24]. By using specific Unc5b blocking antibody, we found that Unc5b did not have any effect on Netrin-1-mediated adhesion (data not shown). Integrin receptors are involved in multiple process including cell adhesion [28, 29]. By using blocking antibody against integrin receptor *α*6*β*4, we observed that *α*6*β*4 blocking antibody could partially block Netrin-1-mediated cell adhesion, suggesting that integrin receptor *α*6*β*4 may be involved in Netrin-1-mediated cell adhesion. Furthermore, we tried to assess whether Netrin-1 had synergistic effects on fibronectin-mediated cell migration. When Netrin-1 was added to the lower chamber of the Transwell, it can promote fibronectin-mediated cell migration (Figure 3(d)).

### 3.4. Netrin-1 Promotes Chemotactic Effects of CXCL12 on Thymocytes

**Figure 4:**
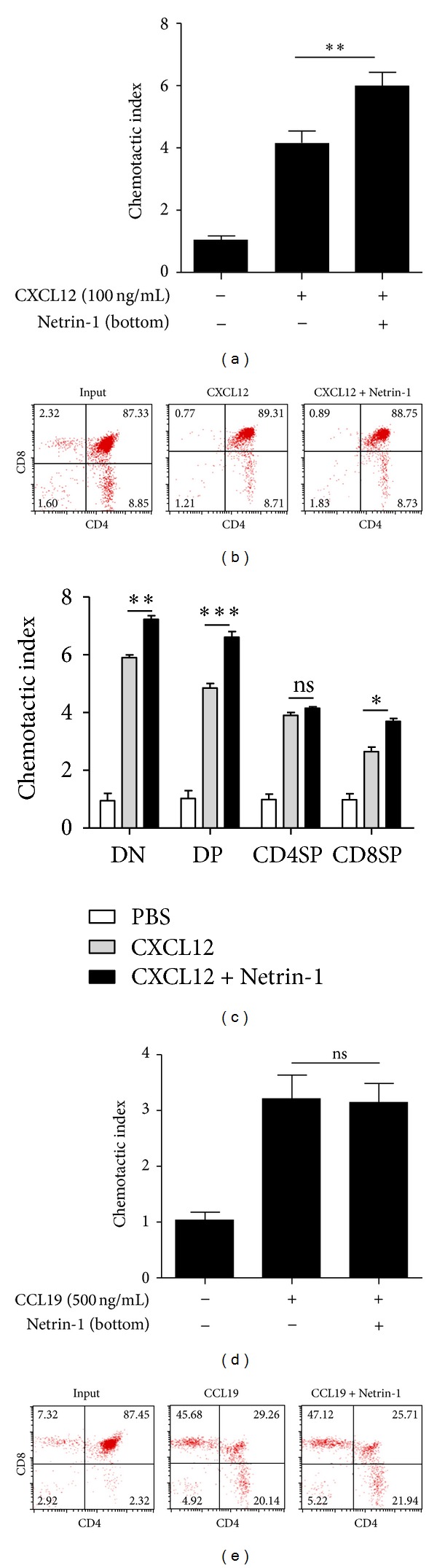
Netrin-1 up-regulation of CXCL12-driven mice thymocyte migration. (a) Netrin-1 partially augmented the chemoattraction of mice thymocytes induced by CXCL12 in the lower chamber. (b) Cells migrated to the bottom well from (a) were harvested and stained with anti-CD4 and anti-CD8. The numbers in the panels are % of gated populations. (c) The effect of Netrin-1 on CXCL12 could be seen in total thymocytes, as well as in DN, DP, and CD8SP subsets. (d) Netrin-1 had no effect on total thymocytes migration induced by CCL19. (e) Cells migrated to the bottom well from (d) were harvested and stained with anti-CD4 and anti-CD8. The numbers in the panels are % of gated populations. Experiments are representatives of at least three independent experiments. ns: not significant; *P < 0.05; **P < 0.01; ***P < 0.001.

In the nervous system, it has been shown that Netrin-1 can mediate chemoattraction or chemorepulsion on target cells. It prompted us to investigate whether Netrin-1 was also involved in thymocyte migration. When Netrin-1 alone was put in the upper or lower chamber, it did not show any effects on thymocyte migration (Figure 3(d)). It is well known that CXCL12 (SDF-1*α*) is one of the most crucial chemokines that drive thymocyte migration. When applying Netrin-1 into the lower chamber of a Transwell device, it had synergistic effects on CXCL12-mediated total thymocyte chemotaxis (Figure 4(a)). However, it had no apparent effects on the phenotype of the thymocytes in the lower chamber which was migrated cells (Figure 4(b)). When different subsets of thymocytes were used to be responsive to CXCL12, copresence of Netrin-1 mainly induced an increase in CXCL12-mediated DN, DP, and CD8SP thymocyte migration (Figure 4(c)).

In similar assays performed with another chemokine, CCL19, Netrin-1 did not show any effects on CCL19-mediated thymocyte chemotaxis and the phenotype of migrated cells (Figures 4(d) and 4(e)), suggesting the specificity of the regulating effects of Netrin-1 on chemokines. These results suggest that Netrin-1 may play a crucial role in thymus development by regulating CXCL12-mediated thymocyte migration.

## 4. Discussion

The thymus is an organ for T-cells differentiation and selection. Developing thymocytes travel within the thymus through appropriate migration to undergo positive and negative selection in the cortex and medulla of the thymus, respectively. In the process of travelling, there are several events for thymocytes to adhere to their surrounding ECM and various types of stromal cells within the thymus. Integrin receptors, binding to ECM, could modulate the strength of cell-cell contact [30]. Accordingly, it is conceivable that interactions between ECM proteins and their corresponding receptors may lead to initiation of adhesion and chemoattraction. In this respect, molecules being involved in the adhesion of thymocytes to ECM and the thymocyte migration to multiple environments of the thymus are still not well defined.

Axon guidance molecules such as Semaphorins and Ephrins have been reported to function in the thymocyte development or migration [31, 32]. Previously, another known axon guidance molecule Netrin-1 has been identified as a negative regulator of the migration of monocytes, neutrophils, and lymphocytes via its receptor, Unc5b [24]. Given its role in the migration of peripheral lymphocytes, it prompted us to probe the expression of Netrin-1 and its receptors in the thymus and further investigate its possible effects on thymocyte behavior such as adhesion and migration. Our data showed that, in unstimulated conditions, Netrin-1 was mainly expressed in thymic stromal cells rather than thymocytes. However, anti-CD3 stimulation could upregulate the expression of Netrin-1 in thymocytes. IL-7 can also upregulate its expression in thymocytes. TEC constitutively secrete IL-7, which is crucial for progression of very immature thymocytes. Anti-CD3 stimulation can mimic the signal of TCR activation and cell-cell interaction. Both anti-CD3 and IL-7 stimulation can upregulate the expression of Netrin-1 in thymocytes significantly, suggesting that Netrin-1 expression in thymocytes is tightly regulated through interactions with thymic stroma. The most apparent upregulation occurred in DP thymocytes, implying that Netrin-1 may be involved in the correct localization of DP thymocytes and is essential for the proper selection of DP thymocytes by thymic stromal cells. Immunohistochemistry staining showed that Netrin-1 was broadly expressed in the thymus, mainly colocalized with a cTEC marker (cytokeratin 8). Furthermore, we also examined the expression of multiple candidate receptors for Netrin-1 and found that Unc5b and integrin *α*6*β*4 represented the main receptors in the thymocytes. Therefore, expression profiles indicate that Netrin-1 and its receptors are highly expressed in the thymus and suggest that Netrin-1 signaling may play a pivotal role during thymocyte adhesion or migration.

Netrin-1 is a laminin-related molecule and plays an adhesive role in the building of nonneuronal structures in organs, such as the pancreas and the mammary gland [33, 34]. We aimed to answer whether Netrin-1 functions like other extracellular matrix molecules in the thymus. Fibronectin is the most important ECM in thymocyte development [35, 36]. Like fibronectin, plate-coated Netrin-1 also adhered thymocytes and this effect was comparable to or even stronger than the effects mediated by fibronectin. Moreover, like other ECM, Netrin-1-mediated adhesion was integrin receptor *α*6*β*4 dependent.

We then tried to find whether Netrin-1 alone had an effect on thymocyte migration. No matter being in the upper or lower chamber of the Transwell, Netrin-1, itself, had no effects on thymocyte migration. This is inconsistent with the data obtained in periphery [24, 25]. The main reasons for this discrepancy possibly result from that Netrin-1 may have different functions in different contexts. It is well known that ECM collaborate with chemokines to exert their functions [37]. Then, we investigated whether Netrin-1 had promotive or antagonizing effects on chemokine-induced thymocyte migration. Two main chemokines CXCL12 and CCL19 were tested. We found that Netrin-1, acting as a chemoattractive stimulus, specifically promoted CXCL12-induced chemotaxis. This is consistent with already published data that laminin can remarkably synergize CXCL12-induced chemotaxis [38]. However, the signaling pathways mediated by Netrin-1 in this process remain to be determined.

In conclusion, we report for the first time that Netrin-1 is expressed in the thymus by both thymic stromal cells and thymocytes. It can mediate cell adhesion and potentiate the chemotaxis for chemokine CXCL12. These data suggest that Netrin-1 may play an important role in thymocyte differentiation, by mediating the interaction between thymocytes and ECM proteins or chemokines through adhesion and chemotaxis mechanisms. Our findings support the implication of the role of Netrin-1 in the modulation of crucial events contributing to thymocyte adhesion and migration, thus promoting the interaction between developing thymocytes and stromal cells and favoring the maturation of intrathymic T cells. As Netrin-1 is also known to control cell death and survival in numerous cancers, we will be further interested in whether Netrin-1 regulates thymocyte survival.
